# P-1542. Itaconic Acid Exerts Antifungal Properties in Macrophages Against Cryptococcus neoformans

**DOI:** 10.1093/ofid/ofaf695.1723

**Published:** 2026-01-11

**Authors:** Kyungyoon Yoo, Alka Kumari, Camila Boniche-Alfaro, Ulrike Beutling, Mark Brönstrup, Frank Pessler, Bettina F Fries

**Affiliations:** Renaissance School of Medicine at Stony Brook University, Stony Brook, NY; Stony Brook University, Stony Brook, New York; Stony Brook University, Stony Brook, New York; Helmholtz Centre for Infection Research, Braunschweig, Niedersachsen, Germany; Helmholtz Centre for Infection Research, Braunschweig, Niedersachsen, Germany; TWINCORE Centre for Experimental and Clinical Infection Research, Hannover, Niedersachsen, Germany; Renaissance School of Medicine at Stony Brook University, Stony Brook, NY

## Abstract

**Background:**

*Cryptococcus neoformans (*CN*)* is an opportunistic fungal pathogen that causes more than 180,000 deaths annually. From primary infection by inhalation, fungal cells can disseminate to the central nervous system and cause cryptococcal meningitis, particularly in immunocompromised populations. Estimated one-year mortality for patients receiving care can be as high as 70%, suggesting that current antifungal treatments are inadequate. Macrophages are primary effector cells for cryptococcal elimination from the host, and the interaction between macrophages and CN can dictate the outcome of cryptococcal infection. Macrophages can control the growth of intracellular pathogens, which involves the production of itaconic acid (IA), an immunomodulatory metabolite produced within the TCA cycle. Although IA and IA derivatives have been shown to have antimicrobial properties through direct antibacterial effects and by enhancing intracellular killing, it is not known whether these compounds can alter the outcome of cryptococcal infection.

**Methods:**

Human and mouse macrophages were infected with antibody/serum opsonized CN. mRNA expression of *Acod1* was measured by RT-qPCR and phagocytic killing by macrophages was determined by quantifying the CFU over time. IA uptake into CN cells was measured by LC-MS, and inhibition of CN growth by IA in axenic culture was determined by tracking the optical density. *Galleria mellonella* and C57BL/6 mice were injected with IA and its derivatives to determine the *in vivo* effects of IA during CN infection.

**Results:**

CN infection induced *Acod1* expression in human and mouse macrophages. Furthermore, addition of exogenous IA and its derivatives improved phagocytic killing, and inhibition of IA synthesis decreased killing of CN cells by macrophages. While CN did not endogenously produce IA, CN cells took up IA which restricted fungal growth in a glucose/pH-dependent manner. Lastly, IA and its analogues improve survival in *Galleria* waxworm infection model and reduce early lung and late brain fungal burden in a mouse infection model.

**Conclusion:**

Our study elucidates the role of macrophage-derived and exogenously administered IA in controlling cryptococcal infection and will help guide the development of host-directed antifungal therapy to treat CN infection.

**Disclosures:**

All Authors: No reported disclosures

